# Bioactive Molecule Prediction Using Extreme Gradient Boosting

**DOI:** 10.3390/molecules21080983

**Published:** 2016-07-28

**Authors:** Ismail Babajide Mustapha, Faisal Saeed

**Affiliations:** 1 UTM Big Data Centre, Ibnu Sina Institute for Scientific and Industrial Research, Universiti Teknologi Malaysia, Skudai, Johor 81310, Malaysia; bmismail2@live.utm.my; 2 Information Systems Department, Faculty of Computing, Universiti Teknologi Malaysia, Skudai, Johor 81310, Malaysia

**Keywords:** biological data, drug discovery, virtual screening, prediction of biological activity

## Abstract

Following the explosive growth in chemical and biological data, the shift from traditional methods of drug discovery to computer-aided means has made data mining and machine learning methods integral parts of today’s drug discovery process. In this paper, extreme gradient boosting (Xgboost), which is an ensemble of Classification and Regression Tree (CART) and a variant of the Gradient Boosting Machine, was investigated for the prediction of biological activity based on quantitative description of the compound’s molecular structure. Seven datasets, well known in the literature were used in this paper and experimental results show that Xgboost can outperform machine learning algorithms like Random Forest (RF), Support Vector Machines (LSVM), Radial Basis Function Neural Network (RBFN) and Naïve Bayes (NB) for the prediction of biological activities. In addition to its ability to detect minority activity classes in highly imbalanced datasets, it showed remarkable performance on both high and low diversity datasets.

## 1. Introduction

Recent advancement in technology has been crucial to the explosive growth in the amount of chemical and biological data available in the public domain. Hence, data driven drug discovery and development process has attracted increased research interest in the last decade with a view not only to design and analyze but apply effective learning methodologies to the rapidly growing data. By leveraging one of the important principles of chemical/molecular similarity [1], where similar biological activities and properties are expected of structurally similar compounds, approaches to drug design through screening of large chemical databases have increased over the years. Virtual Screening (VS), the use of computational approaches and tools through the search of large databases for target or activity prediction, has notably witnessed a shift in trend from the traditional similarity searching, through reference compounds, to the use of machine learning tools to learn from the massive big data by training and prediction of unknown activity. In particular, the compound classification, in which compound label prediction is based on knowledge acquired from a training set, has gained increased research interest and many machine learning tools have been proposed to exploit the increasing big data in drug discovery. Support Vector Machines (SVM) [2,3], DT [4], Random Forest [5], K Nearest Neighbors (K-NN) [6], Naïve Bayes Classifier [7] and Artificial Neural Networks (ANN) [8] are some of the most popular machine learning methods used for activity prediction in compound classification [9].

Despite records of successful application of these methods in cheminformatics and computer aided drug discovery, each method has its peculiar shortcomings and practical constraints; such as predictive accuracy, robustness to high dimensionality and irrelevant descriptors, model interpretability, and computational efficiency, that hinders its optimal performance. For example, DT is a method that performs fairly well when it comes to most of the afore-stated criteria; however, its low predictive accuracy has inspired methods involving an ensemble of trees to improve this shortcoming. One of such efforts produced Random Forest which has been shown to be a reliable machine learning tool for compound classification as reported in [5]. In the same vein, while bearing in mind the No free Lunch Theorem [10]; that there is no best algorithm for all problems, we present herein the findings on another impressive ensemble of tree method called Extreme Gradient Boosting (Xgboost) for bioactive molecule prediction.

Xgboost is an efficient and scalable variant of the Gradient Boosting Machine (GBM) [11] which has been a winning tool for several Machine learning competitions [12,13] in recent years due to its features such as ease of use, ease of parallelization and impressive predictive accuracy. In addition to the obvious fact that alternative approaches to target prediction gives a wider perspective of the data rather than a single approach [14], we show in this paper that Xgboost not only produces comparable or even better predictive accuracy than the state of art in bioactivity prediction, but possess the intrinsic ability to handle the highly diverse and complex feature space of descriptors, especially in situations where the class distribution is highly imbalanced.

## 2. Methods

### 2.1. Tree Ensemble

As described by Chen and Guestrin [15], Xgboost is an ensemble of *K* Classification and Regression Trees (CART) {T_1_(*x_i_*, *y_i_*)…..T*_K_*(*x_i_*, *y_i_*)} where *x_i_* is the given training set of descriptors associated with a molecule to predict the class label, *y_i_*. Given that a CART assigns a real score to each leaves (outcome or target), the prediction scores for individual CART is summed up to get the final score and evaluated through *K* additive functions, as shown in Equation (1):

(1) $${\overset{\wedge }{y}}_{i}=\sum_{k=1}^{K}f_{k}(x_{i}),f_{k}\in F$$

where *f_k_* represents an independent tree structure with leaf scores and *F* is the space of all CART. The regularized objective to optimize is given by Equation (2):

(2) $$Obj(\Theta )=\sum_{i}^{n}l(y_{i},{\overset{\wedge }{y}}_{i})+\sum_{k}^{K}\Omega (f_{k})$$

The first term is a differentiable loss function, *l*, which measures the difference between the predicted $\hat{y}$ and the target *y_i_*. The second is a regularization term Ω which penalizes the complexity of the model to avoid over-fitting. It is given by $\Omega \left(f\right)=\gamma T+\frac{1}{2}\lambda \sum_{j-1}^{T}w_{j}^{2}$ Where *T* and *w* are the number of leaves and the score on each leaf respectively. γ and λ are constants to control the degree of regularization. Apart from the use of regularization, shrinkage and descriptor subsampling are two additional techniques used to prevent overfitting [15].

Training. For a training dataset of molecules with vectors of descriptors and their corresponding class labels or (e.g., active/inactive) or activity of interest, the training procedure in Xgboost is summarized as follows;

- For each descriptor,
  - Sort the numbers
  - Scan the best splitting point (lowest gain)
- Choose the descriptor with the best splitting point that optimizes the training objective
- Continue splitting (as in (i) and (ii)) until the specified maximum tree depth is reached
- Assign prediction score to the leaves and prune all negative nodes (nodes with negative gains) in a bottom-up order
- Repeat the above steps in an additive manner until the specified number of rounds (trees K) is reached.

Since additive training is used, the prediction $\hat{y}$ at step *t* expressed as

(3) $${{\overset{\wedge }{y}}_{i}}^{\left(t\right)}=\sum_{k=1}^{K}f_{k}(x_{i})={{\overset{\wedge }{y}}_{i}}^{\left(t-1\right)}+f_{t}(x_{i})$$

And Equation (2) can be written as

(4) $$Obj{\left(\Theta \right)}^{\left(t\right)}=\sum_{i}^{n}l(y_{i},{{\overset{\wedge }{y}}_{i}}^{\left(t-1\right)}+f_{t}(x_{i}))+\Omega (f_{t})$$

And more generally by taking the Taylors expansion of the loss function to the second order

(5) $$Obj{\left(\Theta \right)}^{\left(t\right)}=\sum_{i=1}^{n}[l(y_{i},{{\overset{\wedge }{y}}_{i}}^{\left(t-1\right)})+g_{i}f_{t}(x_{i})+\frac{1}{2}h_{i}f_{t}^{2}(x_{i})+\Omega (f_{t})$$

where $g_{i}$ = $\partial_{{{\hat{y}}_{i}}^{\left(t-1\right)}}l(y_{i},{{\hat{y}}_{i}}^{\left(t-1\right)})$ and $h_{i}$ = ${\partial^{2}}_{{{\hat{y}}_{i}}^{\left(t-1\right)}}l(y_{i},{{\hat{y}}_{i}}^{\left(t-1\right)})$ are respectively first and second order statistics on the loss function. A simplified objective function without constants at step *t* is as follows

(6) $$Obj{\left(\Theta \right)}^{\left(t\right)}=\sum_{i=1}^{n}[g_{i}f_{t}(x_{i})+\frac{1}{2}h_{i}f_{t}^{2}(x_{i})]+\Omega (f_{t})$$

The objective function can be written by expanding the regularization term as

(7) $$\begin{array}{ll} Obj{\left(\Theta \right)}^{\left(t\right)} & =\sum_{i=1}^{n}[g_{i}f_{t}(x_{i})+\frac{1}{2}h_{i}f_{t}^{2}(x_{i})]+\gamma T+\frac{1}{2}\lambda \sum_{j=1}^{T}w_{j}^{2}\\ & =\sum_{j=1}^{T}[(\sum_{i\in I_{j}}g_{i})w_{j}+\frac{1}{2}(\sum_{i\in I_{j}}h_{i}+\lambda )w_{j}^{2}]+\gamma T \end{array}$$

where $I_{j}=\{i|q(x_{i})=j\}$ is the instance set of leaf *j*, for a given structure q(x) the optimal leaf weight, $w_{j}^{*}$, and the optimal objective function which measure how good the structure is are given by Equations (8) and (9) respectively

(8) $$w_{j}^{*}=-\frac{G_{j}}{H_{j}+\lambda }$$

(9) $$Obj^{*}=-\frac{1}{2}\sum_{j=1}^{T}\frac{G_{j}^{2}}{H_{j}+\lambda }+\gamma T$$

where $G_{j}=\sum_{i\in I_{j}}g_{i}$ $G_{j}=\sum_{i\in I_{j}}g_{i}$ and $H_{j}=\sum_{i\in I_{j}}h_{i}$.

Equation (10) is used to score a leaf node during splitting. The first, second and third term of the equation stands for the score on the left, right and the original leaf respectively. Moreover, the final term, γ, is regularization on the additional leaf.

(10) $$Gain=\frac{1}{2}[\frac{G_{L}^{2}}{H_{L}+\lambda }+\frac{G_{R}^{2}}{H_{R}+\lambda }-\frac{{\left(G_{L}+G_{R}\right)}^{2}}{H_{L}+H_{R}+\lambda }]-\gamma$$

### 2.2. Machine Learning Algorithms

The performance of Xgboost was compared with four machine learning algorithms that have been used in the previous studies for activity prediction (Lavecchia 2015):The Support Vector Machine LibSVM (LSVM) [16], Random Forest (RF) [5], Naïve Bayes (NB) [17], and the Radial Basis Function Network (RBFN) [18] Classifiers.

## 3. Experimental Design

### 3.1. Datasets

This work was evaluated on seven carefully selected datasets that have been used to validate fingerprint based molecule classification and activity prediction in the past. A description of COX2 cyclooxygenase-2 inhibitors (COX2) (467 samples), benzodiazepine receptor (BZR) (405 samples) and estrogen receptor (ER) (393 samples) datasets [19,20] is shown in Table 1. The compounds are classified as active or inactive, and divided into training (70%) and validation (30%) sets for the purpose of this work. The table shows the mean pairwise Tanimoto similarity that was calculated based on ECFC_4 across all pairs of molecules for both active and inactive molecules.

The fourth dataset utilized as a part of this study is Directory of Useful Decoys (DUD), which was presented by [21]. Although recently compiled as a benchmark data, its use in virtual screening can be found in [22,23].The decoys for each target have been chosen to fulfill a number of criteria to make them relevant and as unbiased as possible. Only 12 subsets of the DUD with only 704 active compounds were considered and divided into training (70%) and validation (30%) set in this study as shown in Table 2.

The last three datasets (MDDR1-3), selected from the MDL Drug Data Report MDDR [24], have been previously used for LBVS [22,25] and activity prediction [26]. The MDDR data sets contain well defined derivatives and biologically relevant compounds that were converted to Pipeline Pilot’s ECFC_4 fingerprints and folded to give 1024 element fingerprints. A detailed description of each dataset showing the training (70%) and validation (30%) sets, activity classes, number of molecules per class, and their average pairwise Tanimoto similarity across all pairs of molecules is given in Table 3, Table 4 and Table 5. The active molecules for each dataset were used. For instance, the MDDR1 (Table 3) contains a total of 8294 active molecules, which is a mixture of both structurally homogeneous and heterogeneous active molecules (11 classes). The MDDR2 (5083 molecules) and MDDR3 (8568 Molecules) in Table 4 and Table 5 respectively, contain 10 homogeneous activity classes and 10 heterogeneous ones respectively [27].

**Figure 1:**
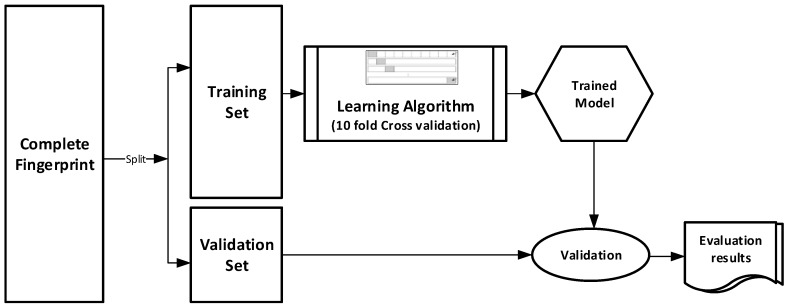
Experimental Design.

The datasets were divided into training (70%) and validation (30%) sets for the purpose of this experiment. Ten-fold cross-validation was used for the Training set. In this cross-validation, the data set was split into 10 parts; 9 were used for training and the remaining 1 was used for testing. This process is repeated 10 times with a different 10th of the dataset used to test the remaining 9 parts during every run of the 10-fold cross validation. Figure 1 pictorially illustrates the various stages involved in the work under study.

### 3.2. Xgboost and Machine Learning Algorithms Parameters

Identifying the optimal parameters for a classifier can be time consuming and tedious and Xgboost is not an exception. This is even more challenging in Xgboost due to the wide range of tuneable parameters for optimal performance; a few of which, using the R [28] implementation of Xgboost, we have restricted our scope to in this work. Thus, by using brute force, we obtained the best performance for Xgboost when eta, gamma, minimum child weight and maximum depth were 0.2, 0.16, 5 and 16 respectively. Where; eta is the step size shrinkage meant to control the learning rate and over-fitting through scaling each tree contribution, gamma is the minimum loss reduction required to make a split, minimum child weight is the minimum sum of instance weight needed in a child and max depth is the maximum depth of a child. Other tree booster parameters like maximum delta step, subsample, column sample and the number of trees to grow per round are left at their default values of 1 respectively. For LSVM, WEKA workbench offers a way to automate the search for optimal parameters. By using grid search, a peak performance with the radial basis kernel was obtained when gamma and cost were 5.01187233627273 × 10^4^ and 20 respectively. RF performed best when the maximum depth of tree was not constrained and the number of iteration set to its default value of 100. The NB classifier achieved best performance when kernel estimator parameter is used instead of normal distribution. For RBFN, we converted numeric attributes to nominal and set the minimum standard deviation to 0.1 to get the best performance.

### 3.3. Evaluation Metrics

The choice of performance evaluation for both model building and validation have been carefully selected from the most commonly used metrics in the literature. The selected evaluation metrics includes the accuracy, area under curve (AUC), sensitivity (SEN), specificity (SPC) and F-measure (F-Sc). The one run definition of AUC (Equation (11)) also known as balanced accuracy which is given by the average of the sum of sensitivity and specificity has been used in this work.

AUC = ((SEN + SPC))/2(11)

while sensitivity (SEN) (Equation (12)) and specificity (SPC) (Equation (13)) show the ability of the model to correctly classify true positive as positive and true negative as negative respectively, AUC simply describes the tradeoff between them.

SEN = tp/(tp + fn)(12)

SPC = tn(tn + fp)(13)

where tp, tn, fp and fn are true positive, true negative, false positive and false negative respectively. In addition to the accuracy (Equation (14)) which is the sum of the correctly classified divided by the total number of classes, F-measure (FSc) (Equation (15)), which is the harmonic mean of precision and recall is included to serve as measure the model’s accuracy.

ACC = ((tp + tn)/(tp + tn + fn + fp))(14)

F Sc = 2 (precision × recall)/(precision + recall)(15)

This work aims to introduce Xgboost for activity prediction through its performance on known datasets in drug discovery. To achieve this aim, the performance of Xgboost was compared with four state of the art machine learning algorithms used in drug discovery based on the afore-stated evaluation metrics. The prediction performances of the different machine learning algorithms on the datasets under study are tabulated in Table 6, Table 7, Table 8, Table 9, Table 10, Table 11 and Table 12. The best values for each metric is shaded.

The classification performance of the MDDR1-3, DUD, COX2, BZR and ER datasets are reported in Table 6, Table 7, Table 8, Table 9, Table 10, Table 11 and Table 12 respectively.

The experimental results on MDDR1-3 Validation datasets (Table 6, Table 7 and Table 8) shows that Xgboost produced the best accuracy, sensitivity, specificity, AUC and F-Sc across all the activity classes compared to the other machine learning methods (RF, LSVM, RBFN and NB) despite the obvious imbalance distribution of activity classes in the most of the datasets. Hence, the Xgboost method performed well for the high diverse dataset (MDDR3), and these results are particularly interesting since the MDDR3 is made up of heterogeneous activity classes which are more challenging for most machine learning algorithms.

For DUD Validation dataset (Table 9), Xgboost and RF produced the best accuracy (0.9471) compared to the other methods. In addition, Xgboost produced the best specificity across all DUD sub datasets. However, NB obtained the best sensitivity, AUC and F-Sc results.

For COX2, ER and BZR Validation datasets (Table 10, Table 11 and Table 12), it is shown that Xgboost performed well and produced the best accuracy and AUC for COX2 and ER datasets. In addition, it obtained the best F-Sc results for COX2 dataset compared to the other state-of-art methods.

Visual inspection of the results shows that Xgboost produced the best accuracy for all used datasets (except for BZR dataset which produced the second best accuracy). While the performance of Xgboost on most activity classes in terms of accuracy and AUC remains the best, it still produces the best average performance across all evaluation metrics. In addition, the good performance of Xgboost is not only restricted to homogenous activity classes since it also performed well on the heterogeneous dataset.

Moreover, a quantitative approach using Kendall W test of concordance was used to rank the effectiveness of all used methods as shown in Table 13. This test shows whether a set of raters make comparable judgments on the ranking of a set of objects. Hence, the XGB, RF, LSVM, RBFN and NB methods were used as the raters, and the accuracy measure (using MDDR1-3, DUD, COX2, BZR and ER datasets respectively) were used as the ranked objects. The outputs of this test are the Kendall coefficient (W) and the associated significance level (p value). In this paper, if the value is significant at a cutoff value of 0.01, then it is possible to give an overall ranking for the methods.

The results of the Kendall analysis for the seven datasets are shown in Table 13. The columns show the evaluation measure, the value of the Kendall coefficient (W), the associated significance level (p value), and the ranking of prediction methods. The overall rankings of the four methods show that Xgboost significantly outperforms the other methods using accuracy measure across all datasets.

## 4. Conclusions

This paper investigated the performance of Xgboost on bioactivity prediction and found out that Xgboost is indeed a robust predictive algorithm. Experimental results show that Xgboost is not only effective as a predictive model for homogeneous dataset but can replicate such effectiveness on structurally heterogeneous dataset. Experimental results show that Xgboost produces an impressive predictive accuracy, ranging from 94.47% accuracy in the heterogeneous data to 98.49% in the homogeneous one. In addition to the obvious fact that Xgboost has been shown in this work to be a good predictive tool for bioactive molecule, we are hopeful that by this Xgboost would be seen as an invaluable addition to already known computational approaches to target prediction and thus leading to a wider perspective of the data rather than a single approach.

## Figures and Tables

**Table 1:** Activity Classes for cyclooxygenase-2 (COX2) estrogen receptor (ER) and benzodiazepine receptor (BZR) Datasets.

Datasets	Number of Compounds				Pairwise Similarity (Mean)	
	Active		Inactive		Active	Inactive
	Training	Validation	Training	Validation		
Cyclooxygenase-2 inhibitors	211	92	116	48	0.687	0.690
Benzodiazepine receptor	214	92	70	29	0.536	0.538
Estrogen receptor	86	55	190	62	0.468	0.456

**Table 2:** Number of Active (Na) compounds for 12 Directory of Useful Decoys (DUD) datasets.

No	Activity Class	Na	
		Training	Validation
1	FGFR1T	90	30
2	FXA	106	40
3	GART	27	13
4	GBP	38	14
5	GR	55	23
6	HIVPR	42	20
7	HIVRT	32	11
8	HMGA	24	11
9	HSP90	24	13
10	MR	10	5
11	NA	35	14
12	PR	22	5

**Table 3:** Activity Classes for MDDR1.

Activity Index	Activity Class	Active Molecules		Pairwise Similarity
		Training	Validation	Mean
31420	renin inhibitors	783	347	0.573
71523	HIV protease inhibitors	535	215	0.446
37110	thrombin inhibitors	561	242	0.419
31432	angiotensin II AT1 antagonists	674	269	0.403
42731	substance P antagonists	859	387	0.339
06233	5HT3 antagonists	530	222	0.351
06245	5HT reuptake inhibitors	257	102	0.345
07701	D2 antagonists	268	127	0.345
06235	5HT1A agonists	589	238	0.343
78374	protein kinase C inhibitors	326	127	0.323
78331	cyclooxygenase inhibitors	427	209	0.268

**Table 4:** Activity Classes for MDDR2.

Activity Index	Activity Class	Active Molecules		Pairwise Similarity
		Training	Validation	Mean
07707	adenosine (A1) agonists	136	71	0.424
07708	adenosine (A2) agonists	119	37	0.484
31420	renin inhibitors	791	339	0.584
42710	monocyclic β-lactams	78	33	0.596
64100	cephalosporins	911	390	0.512
64200	carbacephems	115	43	0.503
64220	carbapenems	732	319	0.414
64300	penicillin	88	38	0.444
65000	antibiotic, macrolide	268	120	0.673
75755	vitamin D analogous	323	132	0.569

**Table 5:** Activity Classes for MDDR3.

Activity Index	Activity Class	Active Molecules		Pairwise Similarity
		Training	Validation	Mean
09249	muscarinic (M1) agonists	620	280	0.257
12455	NMDA receptor antagonists	990	410	0.311
12464	nitric oxide synthase inhibitors	348	157	0.237
31281	dopamine β-hydroxylase inhibitors	76	30	0.324
43210	aldose reductase inhibitors	663	294	0.37
71522	reverse transcriptase inhibitors	501	199	0.311
75721	aromatase inhibitors	444	192	0.318
78331	cyclooxygenase inhibitors	449	187	0.382
78348	phospholipase A2 inhibitors	430	187	0.291
78351	lipoxygenase inhibitors	1478	633	0.365

**Table 6:** Sensitivity, Specificity, Area under Curve, Accuracy and F-measure on MDDR1 Dataset.

ML Algorithm	Training					Validation				
	SEN	SPC	AUC	ACC	F-Sc	SEN	SPC	AUC	ACC	F-Sc
XGB	0.9484	0.9958	0.9721	0.9575	0.9830	0.9579	0.9960	0.9769	0.9594	0.9536
RF	0.9474	0.9963	0.9718	0.9621	0.9514	0.9502	0.9957	0.9730	0.9590	0.9525
LSVM	0.9258	0.9943	0.9600	0.9425	0.9264	0.9357	0.9948	0.9653	0.9497	0.9371
RBFN	0.7566	0.9773	0.8670	0.7719	0.7451	0.7751	0.9777	0.8764	0.7746	0.7553
NB	0.7648	0.9781	0.8715	0.7826	0.7578	0.7488	0.9762	0.8625	0.7626	0.7383

**Table 7:** Sensitivity, Specificity, Area under Curve, Accuracy and F-measure on MDDR2 Dataset.

ML Algorithm	Training					Validation				
	SEN	SPC	AUC	ACC	F-Sc	SEN	SPC	AUC	ACC	F-Sc
XGB	0.9779	0.9981	0.9880	0.9834	0.9689	0.9820	0.9983	0.9902	0.9849	0.9673
RF	0.9562	0.9979	0.9771	0.9837	0.9689	0.9468	0.9977	0.9723	0.9823	0.9597
LSVM	0.9590	0.9978	0.9784	0.9817	0.9667	0.9436	0.9974	0.9705	0.9790	0.9547
RBFN	0.9507	0.9961	0.9734	9.9646	0.9402	0.9420	0.9960	0.9690	0.9658	0.9312
NB	0.9546	0.9963	0.9755	0.9677	0.9458	0.9401	0.9967	0.9684	0.9724	0.9403

**Table 8:** Sensitivity, Specificity, Area under Curve, Accuracy and F-measure on MDDR3 Dataset.

ML Algorithm	Training					Validation				
	SEN	SPC	AUC	ACC	F-Sc	SEN	SPC	AUC	ACC	F-Sc
XGB	0.9407	0.9937	0.9672	0.9440	0.9348	0.9493	0.9937	0.9715	0.9447	0.9448
RF	0.9209	0.9929	0.9569	94.099	0.9350	0.9316	0.9928	0.9622	0.9397	0.9405
LSVM	0.8800	0.9885	0.93425	90.4651	0.8948	0.8983	0.9902	0.9443	0.9171	0.9120
RBFN	0.7053	0.9643	0.8348	68.0613	0.6597	0.7254	0.9657	0.8456	0.6890	0.6710
NB	0.6803	0.9613	0.8208	65.7276	0.6402	0.6636	0.9594	0.8115	0.6415	0.6211

**Table 9:** Sensitivity, Specificity, Area under Curve, Accuracy and F-measure on DUD Dataset.

ML Algorithm	Training					Validation				
	SEN	SPC	AUC	ACC	F-Sc	SEN	SPC	AUC	ACC	F-Sc
XGB	0.8677	0.9920	0.9298	0.9113	0.8616	0.8569	0.9953	0.9261	0.9471	0.8673
RF	0.8861	0.9935	0.9397	0.9294	0.8908	0.9078	0.9951	0.9515	0.9471	0.9123
LSVM	0.8659	0.9919	0.9289	0.9113	0.8683	0.8738	0.9941	0.9340	0.9375	0.8862
RBFN	0.8228	0.9895	0.9061	0.8871	0.8344	0.8503	0.9931	0.9217	0.9279	0.8537
NB	0.8783	0.9910	0.9346	0.9032	0.8730	0.9177	0.9942	0.9559	0.9375	0.9193

**Table 10:** Sensitivity, Specificity, Area under Curve, Accuracy and F-measure on COX2 Dataset.

ML Algorithm	Training					Validation				
	SEN	SPC	AUC	ACC	F-Sc	SEN	SPC	AUC	ACC	F-Sc
XGB	0.9361	0.9444	0.9403	0.9388	0.9535	0.9570	0.9362	0.9466	0.9500	0.9622
RF	0.9763	0.8879	0.9321	0.9450	0.9581	0.9783	0.8750	0.9266	0.9429	0.9574
LSVM	0.9526	0.9138	0.9332	0.9388	0.9526	0.9565	0.8958	0.9262	0.9357	0.9514
RBFN	0.9293	0.7203	0.8248	0.8379	0.8658	0.9250	0.7000	0.8125	0.8286	0.8605
NB	0.6777	0.9569	0.8173	0.7768	0.7967	0.7065	1.0000	0.8533	0.8071	0.8280

**Table 11:** Sensitivity, Specificity, Area under Curve, Accuracy and F-measure on BZR Dataset.

ML Algorithm	Training					Validation				
	SEN	SPC	AUC	ACC	F-Sc	SEN	SPC	AUC	ACC	F-Sc
XGB	0.9764	0.9028	0.9396	0.9577	0.9718	0.9884	0.8000	0.8942	0.9339	0.9551
RF	0.9720	0.9143	0.9431	0.9577	0.9720	0.9674	0.8966	0.9320	0.9504	0.9674
LSVM	0.9579	0.8714	0.9147	0.9366	0.9579	0.9348	1.0000	0.9674	0.9504	0.9663
RBFN	0.9947	0.7263	0.8605	0.9049	0.9330	1.0000	0.6444	0.8222	0.8678	0.9048
NB	0.9112	0.8571	0.8842	0.8979	0.9308	0.8478	0.9655	0.9067	0.8760	0.9123

**Table 12:** Sensitivity, Specificity, Area under Curve, Accuracy and F-measure on ER Dataset.

ML Algorithm	Training					Validation				
	SEN	SPC	AUC	ACC	F-Sc	SEN	SPC	AUC	ACC	F-Sc
XGB	0.7671	0.8522	0.8097	0.8297	0.7044	0.8837	0.7703	0.8270	0.8120	0.7755
RF	0.6860	0.8895	0.7878	0.8261	0.7108	0.6364	0.8226	0.7295	0.7350	0.6931
LSVM	0.6628	0.9316	0.7972	0.8478	0.7308	0.6727	0.9194	0.7960	0.8034	0.7629
RBFN	0.7089	0.8477	0.7783	0.8080	0.6788	0.8478	0.7746	0.8112	0.8034	0.7723
NB	0.9767	0.6368	0.8068	0.7428	0.7029	0.9818	0.5645	0.7732	0.7607	0.7941

**Table 13:** Rankings of Prediction Methods based on Kendall W Test Using Accuracy Measure.

Measure	W	P	Ranks
Accuracy	0.65	0.001	XGBOOST > RF > LSVM > RBFN > NB
